# Ice algal bloom development on the surface of the Greenland Ice Sheet

**DOI:** 10.1093/femsec/fiy025

**Published:** 2018-02-10

**Authors:** C J Williamson, A M Anesio, J Cook, A Tedstone, E Poniecka, A Holland, D Fagan, M Tranter, M L Yallop

**Affiliations:** 1 Bristol Glaciology Centre, University of Bristol,12 Berkely Square, Bristol, BS8 1SS, UK; 2 School of Biological Sciences, University of Bristol, 24 Tyndall Avenue, Bristol, BS8 1TQ, UK; 3 Department of Geography, The University of Sheffield, Sheffield, S10 2TN, UK; 4 School of Earth and Ocean Sciences, Cardiff University, Main Building, Park Place, Cardiff, CF10 3AT, UK

**Keywords:** Greenland, ice sheet, ice algae, glacier, zygnametophyceae, albedo

## Abstract

It is fundamental to understand the development of Zygnematophycean (Streptophyte) micro-algal blooms within Greenland Ice Sheet (GrIS) supraglacial environments, given their potential to significantly impact both physical (melt) and chemical (carbon and nutrient cycling) surface characteristics. Here, we report on a space-for-time assessment of a GrIS ice algal bloom, achieved by sampling an ∼85 km transect spanning the south-western GrIS bare ice zone during the 2016 ablation season. Cell abundances ranged from 0 to 1.6 × 10^4^ cells ml^−1^, with algal biomass demonstrated to increase in surface ice with time since snow line retreat (R^2^ = 0.73, *P* < 0.05). A suite of light harvesting and photo-protective pigments were quantified across transects (chlorophylls, carotenoids and phenols) and shown to increase in concert with algal biomass. Ice algal communities drove net autotrophy of surface ice, with maximal rates of net production averaging 0.52 ± 0.04 mg C l^−1^ d^−1^, and a total accumulation of 1.306 Gg C (15.82 ± 8.14 kg C km^−2^) predicted for the 2016 ablation season across an 8.24 × 10^4^ km^2^ region of the GrIS. By advancing our understanding of ice algal bloom development, this study marks an important step toward projecting bloom occurrence and impacts into the future.

## Teaser

Space for time assessment of an ice algal bloom occurring in surface ice of the Greenland ice sheet.

## INTRODUCTION

Micro-algal residence within supraglacial environments has been reported from numerous polar and high-altitude locations, including Antarctica (Ling and Seppelt 1990), Alaska (Takeuchi, 2001, 2013; Ganey *et al*. 2017), Siberia (Takeuchi *et al*. 2006, 2015), the Himalayas (Yoshimura, Kohshima and Ohtani 1997) and the Greenland ice sheet (GrIS). For the latter, the presence of micro-algal communities on surface ice has been known since the second half of the 19th century (Nordenskiöld 1872), though only recently have studies reported the potential for wide-spread ‘ice algal’ blooms during summer ablation periods (Uetake *et al*. 2010; Stibal *et al*. 2012; Yallop *et al*. 2012; Lutz *et al*. 2014; Stibal *et al*. 2017a). Distinct from communities associated with snow-pack (e.g. Chlorophyta) and cryoconite (e.g. Cyanobacteria) habitats (Lutz *et al*. 2014), ice algal assemblages are comprised of few, specialised taxa belonging to the Zygnematophyceae (Streptophyta) (Remias, Holzinger and Lütz 2009; Remias *et al*. 2012a,b). Blooms occur within the upper few centimetres of surface ice when liquid water, photosynthetically active radiation and nutrient resources are available during the ablation season (Yallop *et al*. 2012; Stibal *et al*. 2017a; Stibal, Bradley and Box 2017b), and are manifest through the brownish-greyish colouration they lend to the ice surface, which is often described as dark- or dirty-ice (Yallop *et al*. 2012; Chandler *et al*. 2015; Anesio *et al*. 2017; Musilova *et al*. 2017).

Additional to the typical suite of light harvesting and photo-protective pigments associated with green microalgae (Remias, Holzinger and Lütz 2009; Yallop *et al*. 2012), ice algae are known to produce a purpurogallin-type phenolic pigment that is postulated to provide photo-protection against excessive UV and visible light experienced in supraglacial environments (Remias *et al*. 2012a,b). This pigmentation, coupled with the high abundance of cells achieved during blooms (∼10^4^ cells ml^−1^; Yallop *et al*. 2012; Stibal *et al*. 2017a), has been suggested to be one of the main drivers of albedo reduction (surface darkening) reported from numerous polar regions, including the western margin of the GrIS in the so-called dark zone (Yallop *et al*. 2012; Stibal *et al*. 2017a; Van Den Broeke [Bibr bib4][Bibr bib27]). As albedo has a primary control on surface melt (Box *et al*. 2012), biological-albedo reduction associated with ice algal blooms may have contributed to the accelerating surface run-off apparent from the GrIS since the early 1990s (Yallop *et al*. 2012; Van den Broeke *et al*. 2017; Stibal *et al*. 2017a), a primary driver of global sea level rise (Van den Broeke *et al*. 2017). Yet empirical evidence to fully quantify the role of ice algae in this process is currently lacking (Cook *et al*. 2017; Tedstone *et al*. 2017).

In addition to feedbacks on surface melt, ice algal blooms may also impact carbon and nutrient cycling within supraglacial habitats, with consequences for down-stream ecosystems (Stibal *et al*. 2012). Whilst the greatest microbial activity is commonly associated with cryoconite debris (Anesio *et al*. 2009; Hodson *et al*. 2010), surface ice dominated by ice algae may fix substantially more CO_2_ than cryoconite given its greater spatial extent (Cook *et al*. 2012; Yallop *et al*. 2012; Chandler *et al*. 2015). A modelling approach demonstrated ice algal communities to be the primary contributors to supraglacial carbon fixation, contributing significantly more than cryoconite-associated communities (Cook *et al*. 2012), with accumulation of autochthonous organic carbon demonstrated within net autotrophic, dirty ice habitats (Musilova *et al*. 2017). Labile organic carbon not consumed *in-situ* by secondary production can be exported by meltwater flushing and utilised within downstream ecosystems (Musilova *et al*. 2017; Smith *et al*. 2017).

Given the potential of ice algal blooms to significantly alter both the physical and chemical supraglacial environment, it is thus fundamental to understand how blooms develop in space and time. Despite this, a limited number of studies have assessed ice algal blooms on the GrIS (e.g. Uetake *et al*. 2010; Yallop *et al*. 2012; Stibal *et al*. 2017a), exemplifying the limited information available for ice algae in comparison to other glacial microbial communities (Anesio *et al*. 2017). Yallop *et al*. (2012) reported maximal densities of ice algae to range 9.1–29.5 × 10^4^ cells ml^−1^ at a marginal south-westerly GrIS location, though observed no spatial trends in algal abundance across their 75 km transect. Recently, Stibal *et al*. (2017a) reported point observations of algal loadings for a range of GrIS locations and monitored bloom dynamics over an ablation season in a similar south-westerly location. They demonstrated that increases in algal abundance with time over the ablation period were moderated by rainfall events, determining a mean population doubling time of 5.5 ± 1.7 days (Stibal *et al*. 2017a).

Here, we report on a space-for-time assessment of an ice algal bloom occurring on the south-western GrIS. As the ablation season proceeds, snow line retreat inland from the ice margin exposes increasing amounts of bare ice in which algal blooms can occur. Sampling along a transect perpendicular to the ice margin thus allows us to substitute space (distance inland from the margin) for time (duration since snow line retreat), permitting a quasi-temporal assessment of bloom development. We focus here predominantly on the bare ice zone (see Hodson *et al*. 2010), the most important region in terms of microbial abundance and activity (Stibal *et al*. 2012), using helicopter transects spanning ∼50 km inland from the ice sheet margin toward the accumulation zone. Emphasis was placed on capturing dynamics in algal biomass and pigmentation across transects, given their importance in driving biological-albedo effects (Cook *et al*. 2017). The carbon fixation potential of ice algal assemblages was further characterised and up-scaled using relationships identified between the time since snow line retreat, algal biomass and net production, to provide the first temporally and spatially resolved estimate of organic carbon accumulation in surface ice driven by a GrIS ice algal bloom.

## METHODS

### Site and sampling details

**Figure 1.**
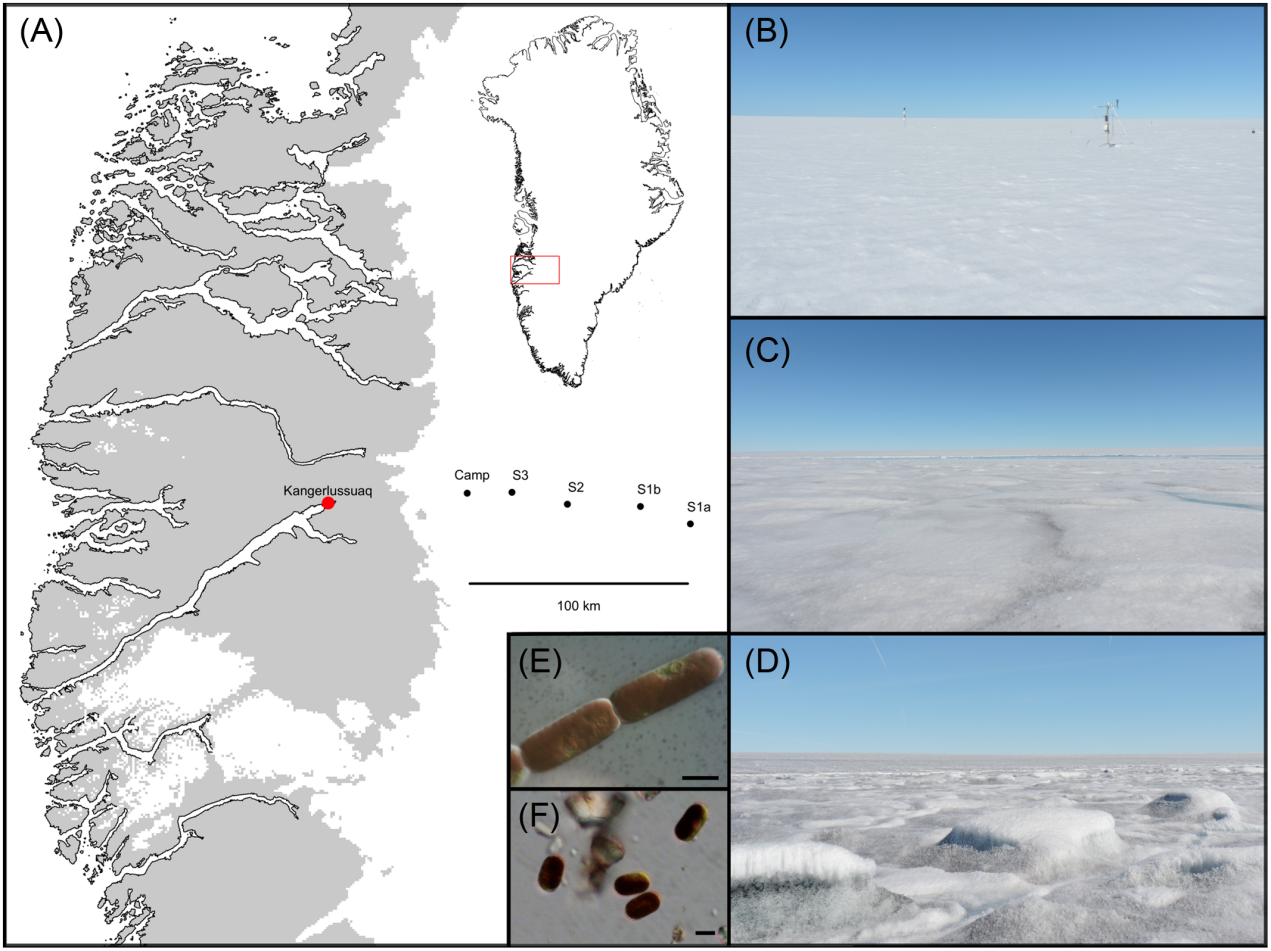
Sampling sites and camp location on the south-western Greenland ice sheet (**A**), with insert showing the relative position of the sampling region within Greenland. Transect 1 (sites S1a, S2 and S3) was performed on the 27th July 2016 (DOY = 209) and transect 2 (sites S1b, S2, S3) on the 5th August 2016 (DOY = 218). Images show the supraglacial surface environment of sites S1a (**B**), S2 (**C**) and S3 (**D**) during transect 1, illustrating the conspicuous increase in surface impurities apparent across transects toward the ice sheet margin. *Ancylonema nordenskiöldii* (**E**) and *Mesotaenium berggrenii* (**F**) were shown by this study to dominate surface ice across transects (scale bars = 10 μm in both cases).

Assessment of an algal bloom occurring in surface ice of the GrIS was achieved using a space-for-time approach by conducting two helicopter transects across ∼85 km of the ablation- to the accumulation-zone of the western ice sheet margin during the 2016 ablation season (Fig. 1 and Table 1). Transects were conducted on the 27th July 2016 (T1, DOY = 209) and the 5th August 2016 (T2, DOY = 218), with three sites examined per transect, ranging from ∼20 to 30 km apart. The most inland sites (S1a and S1b) differed between transects, whilst sites 2 (S2) and 3 (S3) were assessed on both transects. To allow a space-for-time conversion, the duration of time (d) since snow line retreat was determined for each sampling site using MODIS MOD09GA surface reflectance (*R*) data following the approach of Tedstone *et al*. (2017). Briefly, a threshold was applied to daily band 2 (841–876 nm, *R* < 0.6) images and the first day of the melt season at which each sampling site became snow free was identified by applying a 7-day rolling window in which a minimum of 3 days had to be classified as snow-free, 0 as snow-covered and a maximum of 4 days could be cloudy (Table 1). Given the location of site S1a near the equilibrium line, snow line retreat was not predicted at this location before sampling occurred during T1. Across all other sites, the duration since snow line retreat varied inversely with distance from the ice sheet margin (Table 1).

**Table 1.** Site and sampling details.

Site	GPS location	Distance from ice margin	DOY of snow line retreat	Duration since snow line retreat (d)
S1a	67.0003, - 47.0154	∼135 km	213	n.a.
S1b	67.0631, - 47.5433	∼110 km	205	13 (T2)
S2	67.0571, - 48.3064	∼75 km	191	18 (T1)
				27 (T2)
S3	67.0913, - 48.8929	∼50 km	152	57 (T1)
				66 (T2)

Sites S1a and S1b were surveyed during transect 1 (T1, DOY = 209) and transect 2 (T2, DOY = 218), respectively, whereas sites S2 and S3 were surveyed during both transects.

At each site, n = 10 discrete surface areas measuring approximately 20 × 20 × 2 cm (length x width x depth) were sampled in order to constrain ice algal abundance, species diversity and pigmentation. Sample locations were chosen randomly across an area ∼50 × 50 m upwind of the helicopter landing position. Whilst no formal sampling strategy was performed, sampling was conducted to be representative of the surface types present at each site. For each surface sample, a spectral reflectance measurement was taken prior to disturbance of the surface. This was achieved using a Field Spec Pro 3 spectrometer (ASD, USA) with a collimating lens limiting the instantaneous field of view of the fibre optic to 8°, held 30 cm above the sample surface at nadir viewing angle to provide a ground ‘footprint’ of ∼35 cm^2^. The same procedure was used to measure the reflectance of a Spectralon reference panel immediately before and after each measurement of the sample surface to enable the hemispherical conical spectral reflectance factor (HCRF) of the surface to be calculated. Subsequently, the surface was imaged with a scale, and the top 2 cm collected using a metal ice saw and trowel into a sterile Whirl-Pak bag. Sampling tools were washed between samples with MiliQ water and Whirl-Pak bags were immediately placed into the dark and transported to a primary ice camp (∼35 km inland from the ice sheet margin, Fig. 1) within 3 h for processing.

### Sample processing

All samples were melted in the dark over a ∼24 h period at the primary ice camp. Following melting, samples were thoroughly homogenised before sub-sampling. To assess algal cell numbers, species diversity and biovolume, 15 ml of homogenised sample was fixed with 25% glutaraldehyde at 2% final concentration. Samples were stored in the dark under ambient ice sheet temperatures until transport to the University of Bristol, UK, for counting. Counts were performed using a Fuchs-Rosenthal haemocytometer (Lancing, UK) on a Leica DM 2000 epifluorescence microscope with attached MC120 HD microscope camera (Leica, Germany). For those samples containing sufficient cell abundance, a minimum of 300 cells were counted to ensure adequate assessment of assemblage diversity. Imaging for quantification of cell volumes was performed in parallel to counts and measurements of cell diameter and height made using ImageJ software, with cell volume calculated considering ice algal cells as regular cylinders after Hillebrand *et al*. (1999). Cell volumes (μm^3^ cell^−1^) were converted to biomass (pg C cell^−1^) using the relationship of Montagnes *et al*. (1994) between cellular carbon content and volume. Total biomass per sample (μg C ml^−1^) was subsequently calculated as the sum of cell counts (cells ml^−1^) multiplied by average cell biomass (pg C cell^−1^) for each species present.

For characterisation of major chlorophyll and carotenoid pigments, ∼100–200 ml of each homogenised sample was filtered onto a glass fibre filter (GF/F, Whatman, UK) by mild vacuum filtration. Filters were immediately wrapped in foil and frozen in a Biotrek 10 cryoshipper (Statebourne, UK) filled with liquid nitrogen. Filters remained under these conditions during transport to the University of Bristol where upon they were stored at −80°C prior to analysis by high-performance liquid chromatography (HPLC). Following freeze-drying for 24 h, pigments were extracted from filters in 100% acetone containing vitamin E as internal standard. Extractions were analysed using a modified version of the method of Van Heukelem and Thomas (2001), using a c8 column in an Agilent 1100 HPLC equipped with a diode-array detector. Pigments were identified and quantified against analytical standards from DHI and Sigma using both retention time and spectral analysis.

For characterisation of water-soluble pigments, a further ∼100–200 ml of n = 5 samples collected at each site during T2 was filtered onto a separate GF/F filter, frozen and transported back to the University of Bristol, as described above. Filters were subsequently freeze-dried for 24 h, and water-soluble pigments extracted in 5 ml Miliq water following the method of Remias *et al*. (2012b). To remove non-polar constituents (chlorophylls, carotenoids) from the raw extract, a phase separation with *n*-hexane was performed. The aqueous phase was centrifuged and the absorption of the supernatant measured with a WPA Lightwave II UV/visible spectrophotometer (Biochrom, UK) using the wave scan function from 200–750 nm. To provide an estimate of the relative concentration of water-soluble pigments derived from samples, absorbance spectra were normalised to extraction and filtration volumes. Peak height of the dominant peak identified across spectra (λ_335nm_) was subsequently taken as representative of relative concentration and normalised to algal biomass for comparisons between sites.

### Productivity incubations

Ice algal productivity (net photosynthesis and respiration) was assessed at the primary ice camp given the greater duration of time required for incubations and measurements. To assess the relationship between ice algal biomass and productivity, oxygen evolution incubations were performed following the method of Telling *et al*. (2010) with melted (∼24 h in the dark) camp surface ice samples categorised by eye as containing a high, medium or low loading of impurities. Six replicates of each biomass treatment were incubated in 60 ml clear glass biological oxygen demand (BOD) bottles (Wheaton, USA) sealed with ground glass stoppers. Prior to incubations, initial dissolved oxygen concentration and temperature measurements were made using a Microx 4 fibre-optic oxygen meter with attached dipping and temperature probes (PreSens, Germany). Incubations were then sealed and three replicates of each treatment wrapped in foil to create dark conditions for respiration measurements. Bottles were incubated for 24 ± 1 h *in-situ* on the ice surface to maintain ambient temperature and/or light conditions, with a final dissolved oxygen concentration and temperature measurement taken at the end of the incubation period. Daily rates of net production (*NP*, light incubations) or respiration (*R*, dark incubations) (mg O_2_ l^−1^ d^−1^) were calculated as the difference between initial and final oxygen concentrations (mg O_2_ l^−1^), normalised to incubation time (d), as ΔO_2_/Δt. Gross production (*GP*) was subsequently calculated as *NP*—*R*. Data were converted into units of mg C l^−1^ d^−1^ assuming a 1:1 stoichiometry between moles of CO_2_ stored for each mole of O_2_ released, i.e. 0.375 g of C per g O_2_ (Chandler *et al*. 2015). Following incubations, 15 ml subsamples were taken as above for determination of algal cell abundance and biovolume, and calculation of algal biomass as previously detailed. Given the absence of cyanobacteria within incubated samples (determined by microscopy), rates of productivity were assumed representative of the specific contribution of ice algae to organic carbon accumulation/respiration within surface ice. Whilst measured *R* included contributions from both autotrophic and heterotrophic community members, secondary production on the GrIS surface is ∼30-times lower than that of ice algal production (Yallop *et al*. 2012), and thus the contribution of heterotrophs to community respiration was assumed negligible.

To estimate the contribution of ice algal assemblages to net carbon fixation in south-western Greenland, *NP* was modelled over the 2016 ablation period in a ∼8.24 × 10^4^ km^2^ ablation zone area, at 7.5 × 7.5 km resolution. The model area spanned ∼1000 km of latitude along south-western Greenland, intersected by our study transect ∼600 km from the lower limit. Given uncertainties regarding microbial activity within the GrIS marginal zone (Hodson *et al*. 2010; Stibal *et al*. 2012), a ∼7.5 km marginal ice area was excluded from our model region. Pixel by pixel estimates of (i) the day of snow line retreat and (ii) the final day of the ablation period were derived using the regional climate model MARv3.8.1 forced with ERA-Interim (Fettweis *et al*. 2017). The former was estimated as the first day of the year in which the cumulative snowpack depth (*i*th day + the subsequent 4 days) fell to zero metres, with the latter determined as the final day of the year in which cumulative meltwater production (*i*th day + the preceding 4 days) exceeded 3 mm water equivalent. For the duration between these start and end points, daily increases in ice algal abundance were calculated per pixel using the relationship between duration since snow line retreat (d) and ice algal biomass (μg C ml^−1^) determined from field data, and converted to *NP* (mg C l^−1^ d^−1^) using the relationship between ice algal biomass and *NP* determined during the productivity incubations detailed above.

Net carbon assimilation over the 2016 ablation period was thus calculated as the sum of daily *NP* estimates per pixel. To allow conversion from units of volume (l^−1^) to area (km^2^), triplicate 0.5 × 0.5 m surface ice samples containing a low, medium or high biomass of ice algae were sampled at the primary ice camp in an identical fashion to surface sampling described previously, melted over 24 h and the melt volume quantified. Melt volumes did not differ significantly between surface ice of different ice algal loadings, averaging 265.33 ± 16.31 ml across all samples, with 1.061 ± 0.065 l of melt water apparent per m^2^ of sampled ice. The total contribution of ice algal assemblages to net carbon fixation across the south-western GrIS ablation zone was thus calculated as the sum of *NP* per km^2^, multiplied by the extent of our model region.

### Data analysis

All analysis and plotting of data was performed in R v.3.4.1 (R Core Team 2017). Statistical comparisons of ice algal abundance, biovolume and biomass between sites and transects was achieved using analysis of variance (ANOVA) or two-sample t-test comparisons, with post-hoc Tukey HSD analysis applied to all significant ANOVA results. Homogeneity of variance and normality of distribution were tested prior to all parametric analyses, and model assumptions verified by examination of model criticism plots.

## RESULTS AND DISCUSSION

### Extensive algal bloom across the GrIS ablation zone

**Figure 2.**
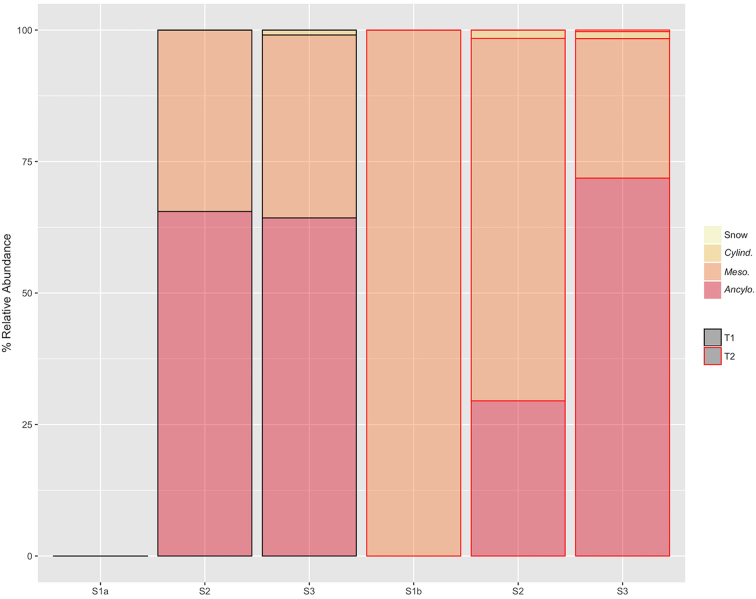
The relative abundance of *Ancylonema nordenskiöldii* (*Ancylo*.), *Mesotaenium berggrenii* (*Meso*.), *Cylindrocystis brebissonii* (*Cylindro*.) and snow-algal resting spores (Snow) apparent across sampling sites during transect 1 (T1) and transect 2 (T2).

Our results revealed the occurrence of a wide-spread algal bloom in surface ice of the south-west GrIS ablation zone, consistent with the heavy colonisation of the so-called dark zone by pigmented autotrophs (Yallop *et al*. 2012; Stibal *et al*. 2017a). Assemblages were invariably populated by ice-algal taxa of the *Zygnematophyceae* (Streptophyte) (Figs 1 and 2), with consistent dominance by *Ancylonema nordenskiöldii* and *Mesotaenium berggrenii* reflecting previous accounts of ice-algal blooms in Russian Siberia (Takeuchi *et al*. 2006; Takeuchi *et al*. 2015), Alaska (Takeuchi 2013) and the south-western GrIS (Yallop *et al*. 2012; Stibal *et al*. 2017a). Microscopy-based findings of the present study were also highly consistent with a molecular-based examination of surface samples from the same locations (Lutz et al. 2018).

Complete absence of algal life at site S1a, particularly species known to bloom in snow-pack environments such as *Chlamydomonas* and *Chloromonas* spp. (Remias, Lutz-Meindl and Lutz 2005), indicated the restriction of blooms of ice-algae to ablation areas in this region of the GrIS. *Ancylonema* and *Mesotaenium* spp. dominated assemblages across all other sites, consistent with their description as ice environment specialists (Takeuchi 2001), and observations of their immediate dominance of glacial ice following snow-line retreat (Takeuchi 2013). *Ancylonema* typically demonstrated the greatest relative abundance (∼65%) during the present study, followed by *Mesotaenium* (∼35%), though deviations from this trend were apparent at sites S1b and S2 during T2 (Fig. 2). *Cylindrocystis brebissonii*, an opportunistic species usually observed at the lowest down-glacier locations (Takeuchi 2013), was recorded here at the lowest relative abundances (∼1%) at sites S2 and S3 only. Data thus add to the growing evidence that GrIS ice algal blooms are consistently dominated by few, specialist taxa, capable of survival and proliferation in extreme cryo-environments (Uetake *et al*. 2010; Yallop *et al*. 2012; Stibal *et al*. 2017a).

**Figure 3.**
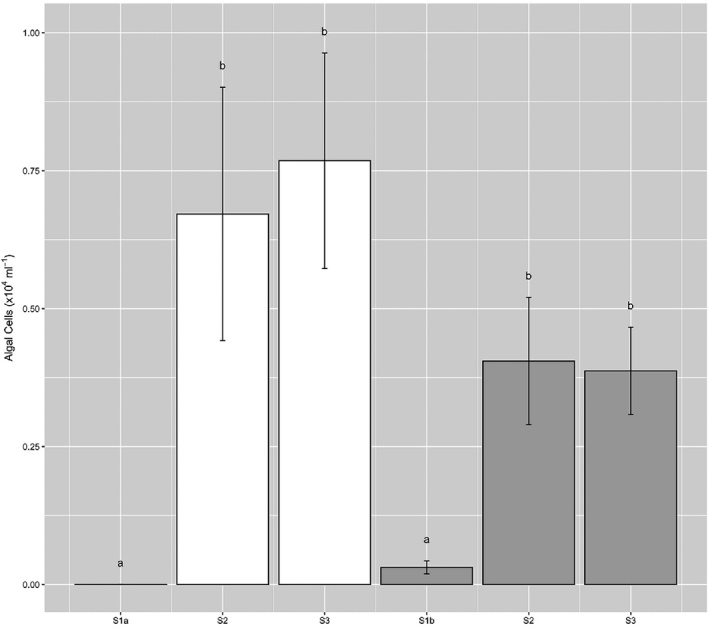
Ice algal cell abundance across sampling sites during transect 1 (white bars) and transect 2 (grey bars) (mean ± SE, n = 10). Lower-case letters denote homogenous subsets determined from 1-way ANOVA analysis of algal abundance ∼ site per transect (Transect 1, *F_2,27_* = 5.88, *P* < 0.01; Transect 2, *F_2,27_* = 6.78, *P* < 0.01). Separate 1-way ANOVA was performed per transect given the assessment of different sites (S1a/S1b) between transects.

**Figure 4.**
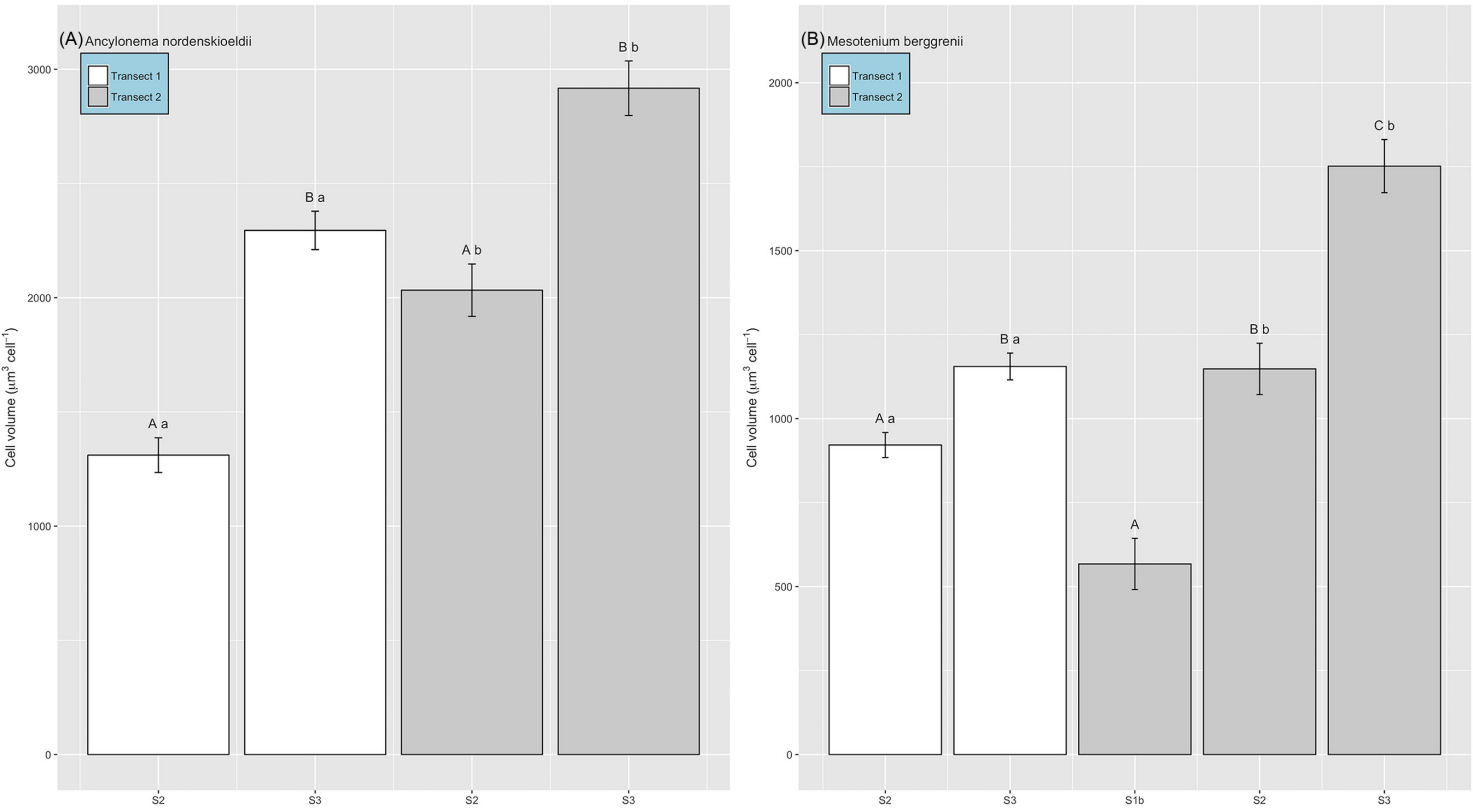
Ice algal biovolume of (**A**) *Ancylonema nordenskiöldii* and (**B**) *Mesotaenium breggrenii* assessed across sampling sites during transect 1 (white bars) and transect 2 (grey bars) (mean ± SE). For *A. nordenskiöldii*, letters denote homogenous subsets determined from 2-way ANOVA of biovolume ∼ site (upper case letters) + transect (lower-case letters) (*F_1,23_* = 14.98 and 20.81, respectively, *P* < 0.001 in both cases). For *M. berggrenii*, given the absence of cells at site S1a during transect 1, biovolume was compared between sites separately across transect 1 (two-sample t-test, *t_14_* = − 3.06, *P* < 0.01) and transect 2 (1-way ANOVA, *F_2,18_* = 16.7, *P* < 0.001), and between transects separately for sites S2 (two-sample t-test, *t_12_* = − 2.24 *P* < 0.05) and S3 (two-sample t-test, *t_17_* = −3.65, *P* < 0.01). Upper case letters denote homogenous subsets in relation to site, and lower-case letters in relation to transect.

**Figure 5.**
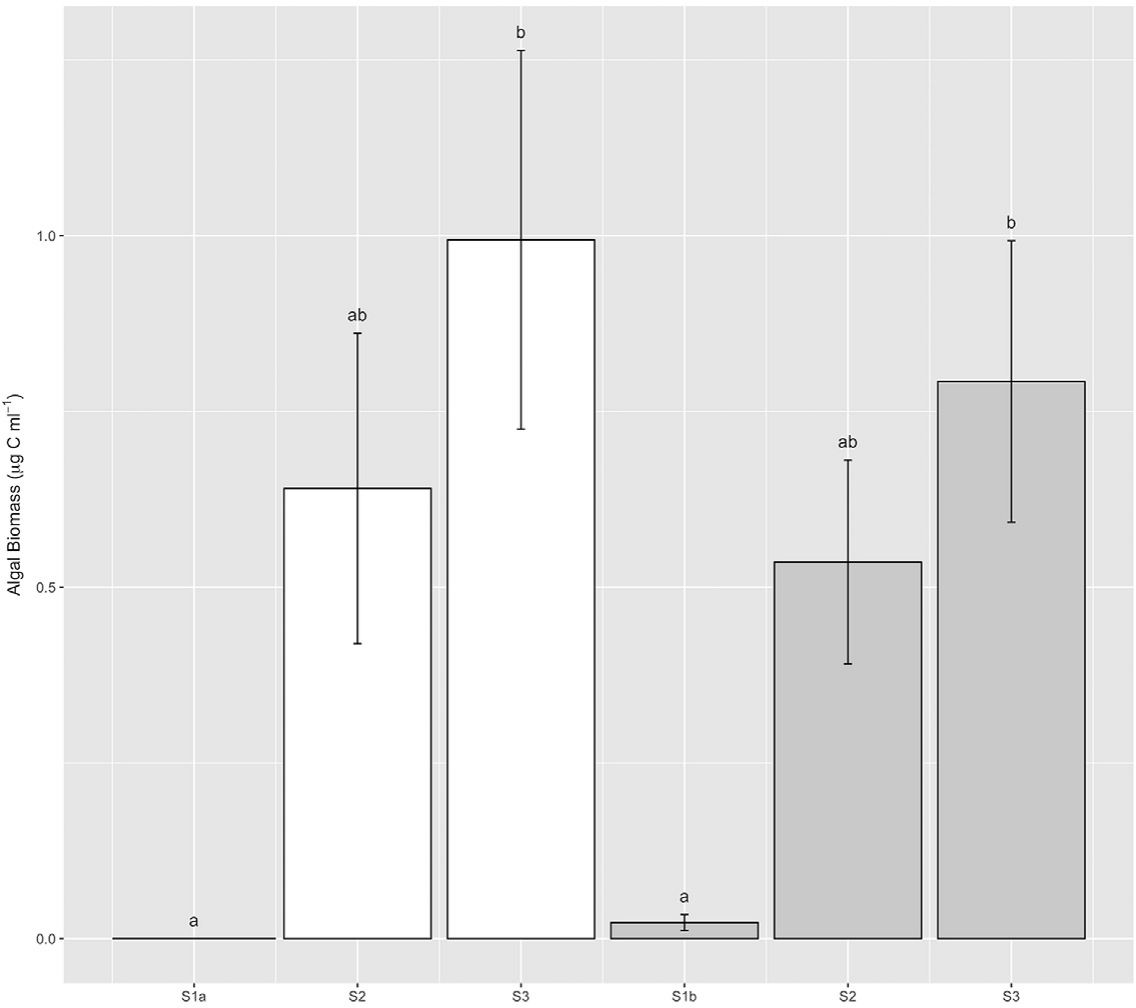
Ice algal biomass within surface ice assessed across sampling sites during transect 1 (white bars) and transect 2 (grey bars) (mean ± SE, n = 10). Lower-case letters denote homogenous subsets determined from 1-way ANOVA analysis of algal biomass ∼ site per transect (Transect 1, *F_2,27_* = 7.07, *P* < 0.01; Transect 2, *F_2,27_* = 8.40, *P*< 0.01). Separate 1-way ANOVA was performed per transect given the assessment of different sites (S1a/S1b) between transects.

Cell abundances ranged from 0 to 1.6 × 10^4^ cells ml^−1^ across all samples (Fig. 3), with maximum densities lower than those reported by Yallop *et al*. (2012) from samples collected closer to the ice-sheet margin (9.1 to 29.5 × 10^4^ cells ml^−1^), though highly comparable to those from site S6 in close proximity to our transect sites (<100 to 8.5 × 10^4^ cells ml^−1^, Stibal *et al*. 2017a). Patterns in algal abundance did not conform to space-for-time expectations of bloom development, i.e. no increase in abundance was evident from S2 to S3 over either transect (39 d time difference), though examination of cell volumes revealed significant increases both across and between transects for the dominant two taxa (Fig. 4). Thus, total algal biomass within surface ice increased across both T1 and T2 toward the ice sheet margin (Fig. 5), revealing a significant linear relationship between average algal biomass within surface ice and time since snow line retreat (R^2^ = 0.73, *P* < 0.05, n = 6). Strong spatial patterning is thus evident in ice algal biomass during blooms in GrIS supraglacial environments, with implications for algal population dynamics.

Dynamism in algal loadings within surface ice was also captured by the present study, with longer-term increases punctuated by shorter-term variability. Significant decrease in algal abundance within sites S2 and S3 over the 9-d period between transects (Fig. 3), though meditated to an extent by increases in cell volume, demonstrated the capacity for significant loss of algal cells from surface environments over short time-scales. Mechanisms for the removal of algal cells from the surface may include mortality and subsequent loss, burial by precipitation and hydrological flushing events (Cameron *et al*. 2017; Stibal *et al*. 2017a; Stibal, Bradley and Box 2017b). For example, Stibal *et al*. (2017a) demonstrated that rainfall events reduce algal abundance in surface ice, such that a significant correlation between the number of days since the last precipitation event and algal abundance was apparent. Whilst the mechanism responsible for cell loss from surface ice is not identifiable here, we contend that the decrease in cell abundance (40%–50% decrease in the 9 days between transect sampling) indicated either a melt or rain driven removal of the heavily colonised surface ice observed during T1, followed by development of a cleaner weathering crust observed during T2. However, assessment of MARv3.8.1 outputs for the period between T1 and T2 failed to highlight melt or precipitation events at either S2 or S3.

### Pigment concentration in surface ice increases as a function of algal biomass

**Figure 6.**
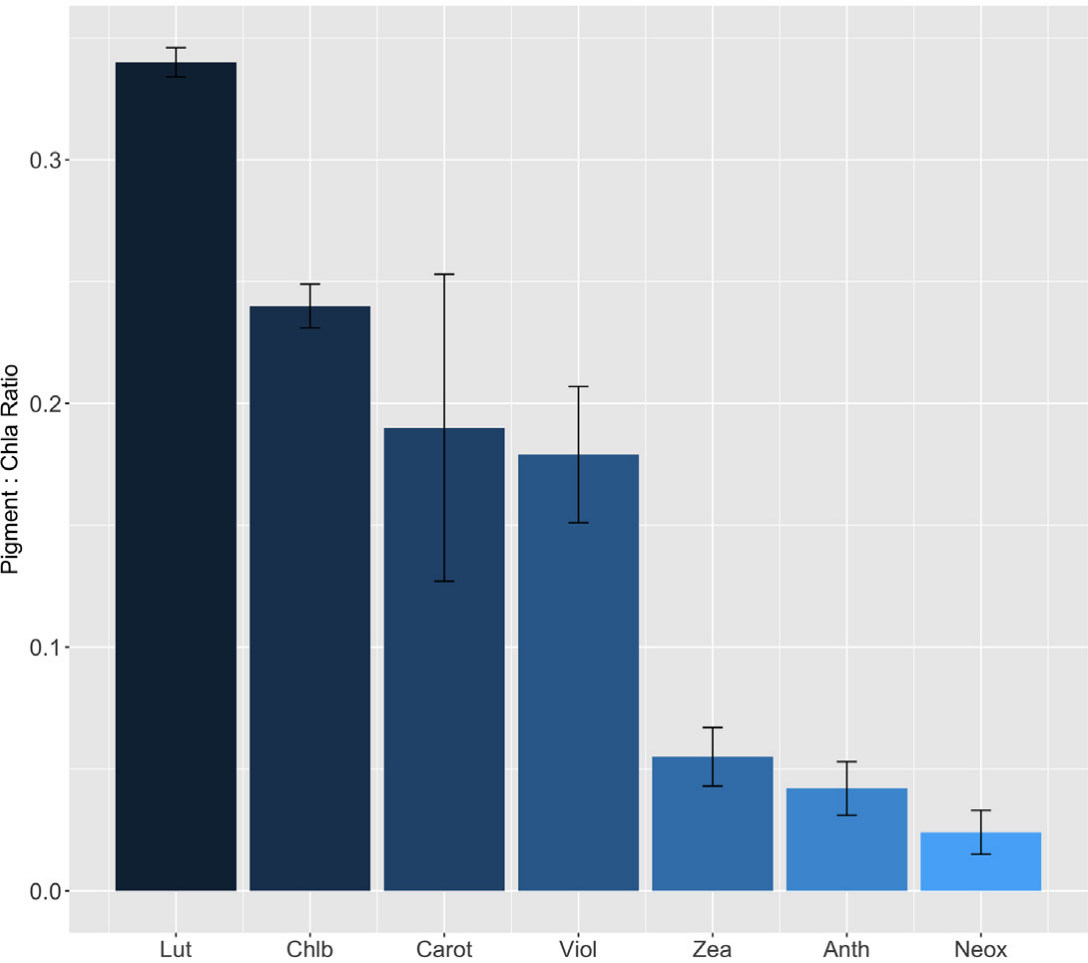
Ice algal chlorophyll and carotenoid pigment ratios relative to chlorophyll *a* (33.02 ± 8.00 fg Chla per pg C^−1^) assessed from surface ice samples collected at S2 during transect 1 (mean ± SE, n = 5). Lut = lutein, Chlb = chlorophyll b, Carot = β-carotene, Viol = violaxanthin, Zea = zeaxanthin, Anth = antheraxanthin, Neox = neoxanthin.

**Figure 7.**
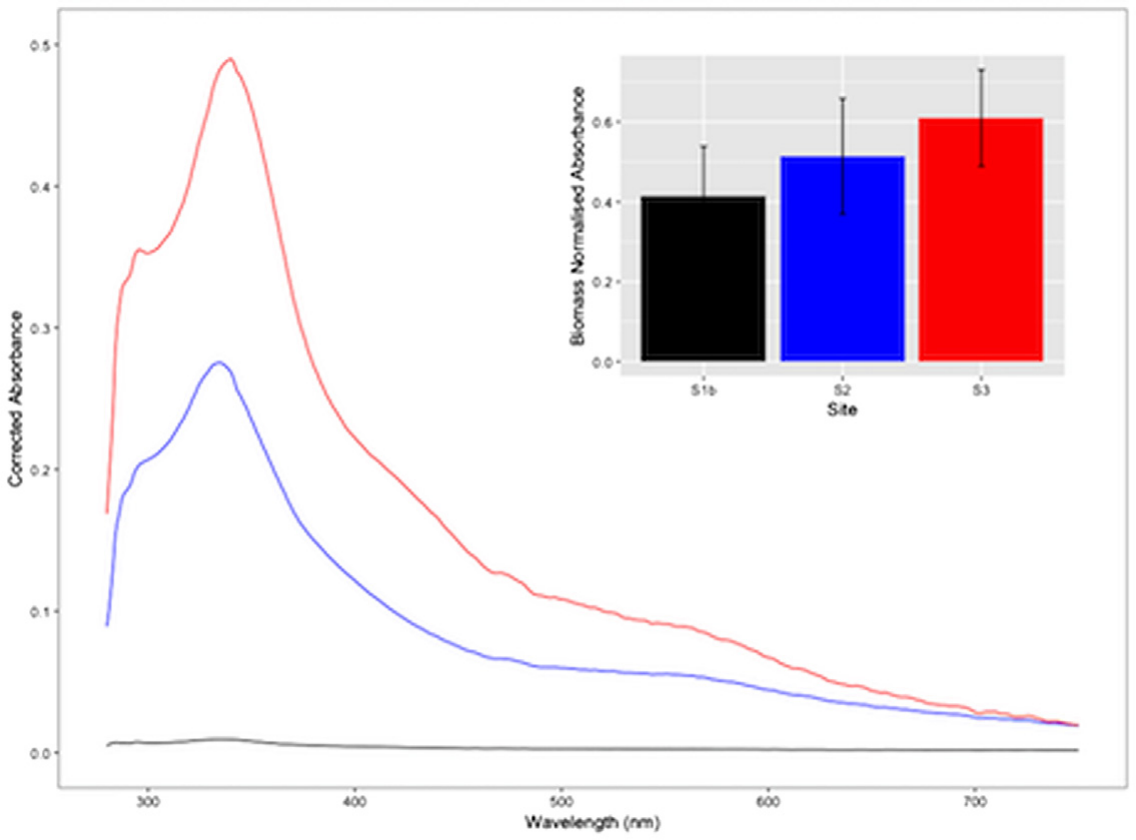
Normalised absorbance spectra of water-soluble pigments derived from surface ice samples at sites S1b (black line), S2 (blue line) and S3 (red line) during transect 2. Insert shows biomass normalised absorbance maxima for the dominant peak (λ_335nm_) identified across spectra (mean ± SE, n = 5).

A suite of light harvesting and photo-protective pigments typical of green micro-algae were identified across samples (Fig. 6), including chlorophylls *a* and *b*, β-carotene, and the xanthophylls antheraxanthin, lutein, neoxanthin, violoxanthin and zeaxanthin. Additionally, abundant secondary pigmentation in the form of UV-VIS absorbing aqueous compounds were extracted from T2 samples (Fig. 7), with highly analogous spectral absorbance signatures to the purpurogallin-derived phenols previously isolated from *A. nordenskioldii* (Remias *et al*. 2012a) and fully characterised from *M. berggrenii* (Remias *et al*. 2012b). Pigments indicative of the presence of oscillatorian cyanobacteria, i.e. scytonemin and echinenone were also recorded within a limited number of surface samples from sites S2 and S3 only, and may have marginally contributed to pigment concentrations determined from these sites. Chlorophyll *a*, the dominant quantifiable pigment in all samples, ranged in concentration from 1.43 ± 0.51 to 21.08 ± 0.95 μg l^−1^, though was not detectable in surface samples from S1a, corroborating the absence of measurable autotrophic life. Pigment profiles thus validated assemblage composition determined by light microscopy, and served to highlight the abundant pigmentation characteristic of the Zygnematophyceae (Holzinger and Pichrtová 2016).

Chlorophyll and carotenoid concentrations varied in concert with algal biomass within surface ice, given that significant differences in biomass-normalised pigment concentrations were absent across and between transects (Table 2). Similarly, biomass-normalised relative concentrations of aqueous extracts did not differ significantly across T2 (Fig. 7), though a general trend of increasing relative concentration was evident across sites S1b to S3, potentially indicating the accumulation of phenol-type pigments at rates greater than biomass increases. Currently, knowledge of the factors that regulate ice-algal pigment accumulation under *in-situ* conditions is lacking (Anesio *et al*. 2017), despite the potential implications for surface albedo and glacier-wide melt processes (Stibal *et al*. 2012; Yallop *et al*. 2012; Cook *et al*. 2017; Tedstone *et al*. 2017; Stibal *et al*. 2017a). Whilst initial studies have characterised the pigments present in dominant ice algal taxa (e.g. Remias *et al*. 2012a,b), a limited number of *in-situ* observations have failed to resolve spatial or temporal patterning in chlorophyll or carotenoid concentrations (Yallop *et al*. 2012; Lutz *et al*. 2014), and no study has examined spatio-temporal dynamics in water-extractable phenols initially documented here. The latter are of particular importance given their presumed facilitation of ice algal dominance in glacial environments via their UV-VIS shading and/or grazer defence qualities (Remias *et al*. 2012a,b, Holzinger and Pichrtová 2016). We demonstrate here that phenol-type pigments are ubiquitous across the extent of ice algal blooms on the GrIS, being present at comparable cellular concentrations from the onset of bloom initiation (i.e. 22 days after snow line retreat) until later bloom stages (66 days after snow line retreat), reinforcing their importance in bloom development.

**Table 2.** Ice algal chlorophyll and carotenoid pigment concentrations normalised to biomass (fg pigment per pg C^−1^) across sites and transects.

Transect	Site	Anth	Chla	Chlb	Carot	Lut	Neox	Viol	Zea
1	S2	1.03 ± 0.01	33.02 ± 8.00	6.38 ± 1.44	4.73 ± 0.29	8.68 ± 1.17	0.80	4.47 ± 0.48	1.35 ± 0.04
	S3	0.91 ± 0.10	24.61 ± 2.78	3.39 ± 0.85	3.59 ± 0.82	5.58 ± 1.07	0.41	3.05 ± 0.56	1.00 ± 0.17
2	S1b	n.d.	41.7 ± 14.90	n.d.	n.d.	7.54 ± 1.96	n.d.	n.d.	n.d.
	S2	1.64 ± 0.27	29.00 ± 3.35	9.27 ± 1.10	4.89 ± 0.57	8.34 ± 0.78	0.65 ± 0.04	3.32 ± 0.40	1.48 ± 0.23
	S3	1.55 ± 0.43	48.08 ± 14.07	8.37 ± 1.27	6.91 ± 0.36	9.13 ± 0.98	0.76 ± 0.12	4.56 ± 0.17	1.52 ± 0.30

Quantifiable pigment concentrations were absent from site S1a during transect 1. n.d. = not detectable, Anth = antheraxanthin, Chla = chlorophyll a, Chlb = chlorophyll b, Carot = β−carotene, Lut = lutein, Neox = neoxanthin, Viol = violaxanthin, Zea = zeaxanthin.

**Figure 8.**
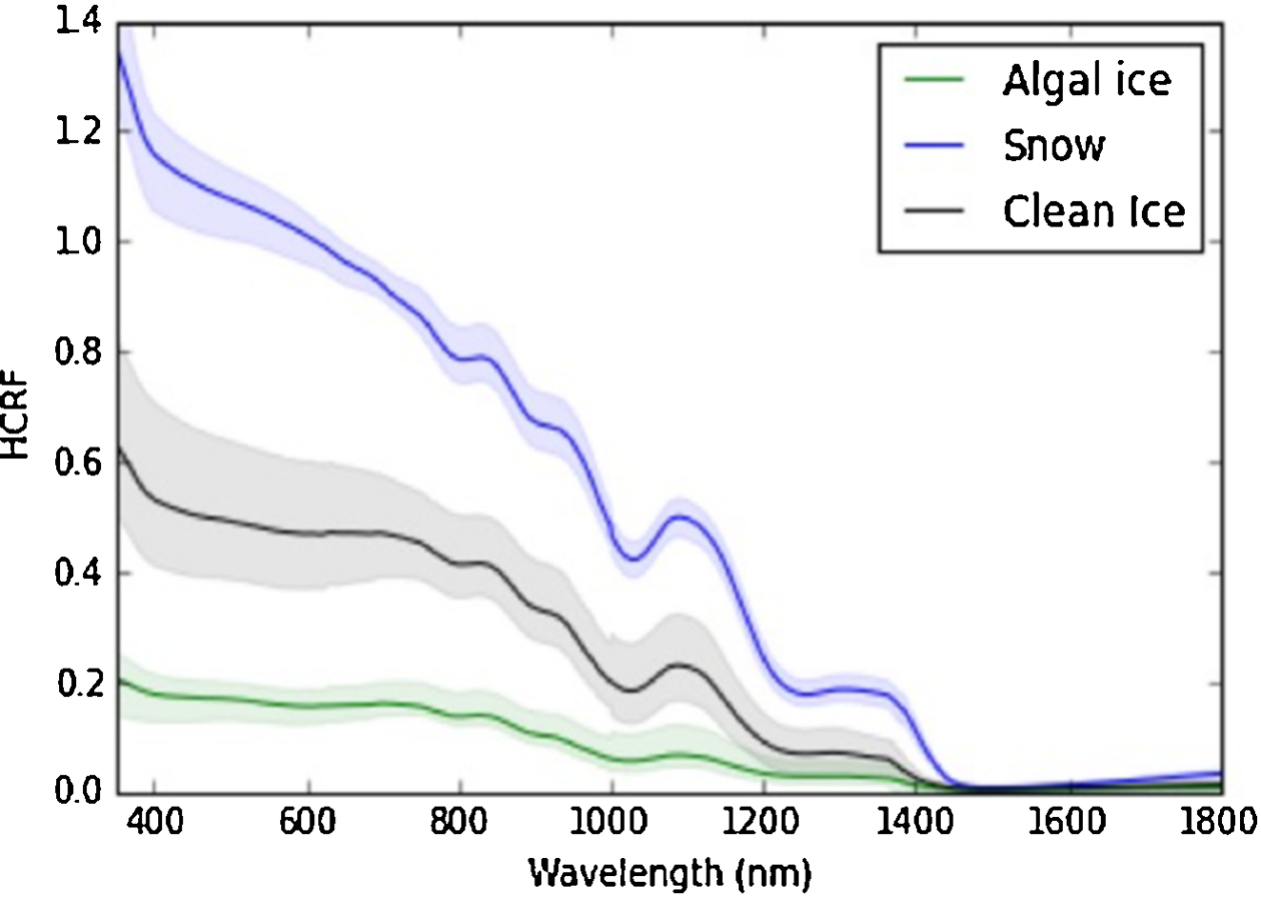
Spectra obtained during transect 1 illustrating the contrasting spectral reflectance of snow (site S1a), clean ice (0 algal cells ml^−1^, Site S3) and algal ice (0.76 ± 0.19 × 10^4^ algal cells ml^−1^, Site S3). HCRF = hemispherical conical reflectance factor, obtained by measuring light reflected from the surface relative to a Spectralon white reference panel. Reference panels can be less reflective than fine grained snow at nadir, explaining reflectance values >100%. Data are presented as a proxy for albedo, though correction for the viewing angle and surface anisotrophy are required to calculate albedo from these data (see Cook *et al*. 2017).

Spectral reflectance measurements made in concert with surface sampling during the present study demonstrated obvious surface darkening for those samples dominated by ice algal assemblages, as compared to clean ice or snow (Fig. 8). These findings are consistent with previous assertions of ice algae as major contributors to albedo reduction across vast areas of the GrIS dark-zone (Yallop *et al*. 2012; Stibal *et al*. 2017a). We emphasise, however, that our ability to directly relate albedo reductions of ice surfaces to ice algal biomass is currently limited by the influence of surface physical characteristics and external factors related to solar and atmospheric effects on ice surface albedo (Cook *et al*. 2017). Additionally, glacial ice can contain concomitant loadings of non-biological impurities whose effect on albedo cannot be readily distinguished from that of biological material (Cook *et al*. 2017). Whilst we have demonstrated that increases in algal biomass are accompanied by increases in primary and secondary pigmentation as ice algal blooms develop on the GrIS, a physical modelling approach has been suggested necessary for disentangling the various biotic and abiotic contributions to surface albedo (Cook *et al*. 2017).

### Ice algal communities drive net autotrophy of the surface ice environment

**Figure 9.**
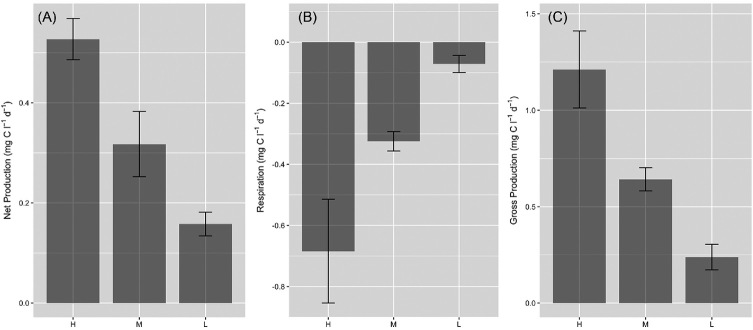
Net production (**A**), respiration (**B**) and gross production (**C**) of surface ice containing a high (H, 3.76 ± 0.56 µg C m^−1^), medium (M, 1.16 ± 0.06 µg C ml^−1^) or low (L, 0.01 ± 0.00 µg C ml^−1^) biomass of ice algae (mean ± SE, n = 3). Lower case letters denote homogenous subsets in relation to biomass for each parameter as determined by 1-way ANOVA (net production, *F_2,6_* = 15.81, *P* < 0.01; respiration *F_2,6_* = 6.56, *P* < 0.05; gross production *F_2,6_* = 11.05, *P* < 0.05). Biomass was significantly different between each biomass category (1-way ANOVA, *F_2,6_* = 33.91, *P* <0.001).

**Figure 10.**
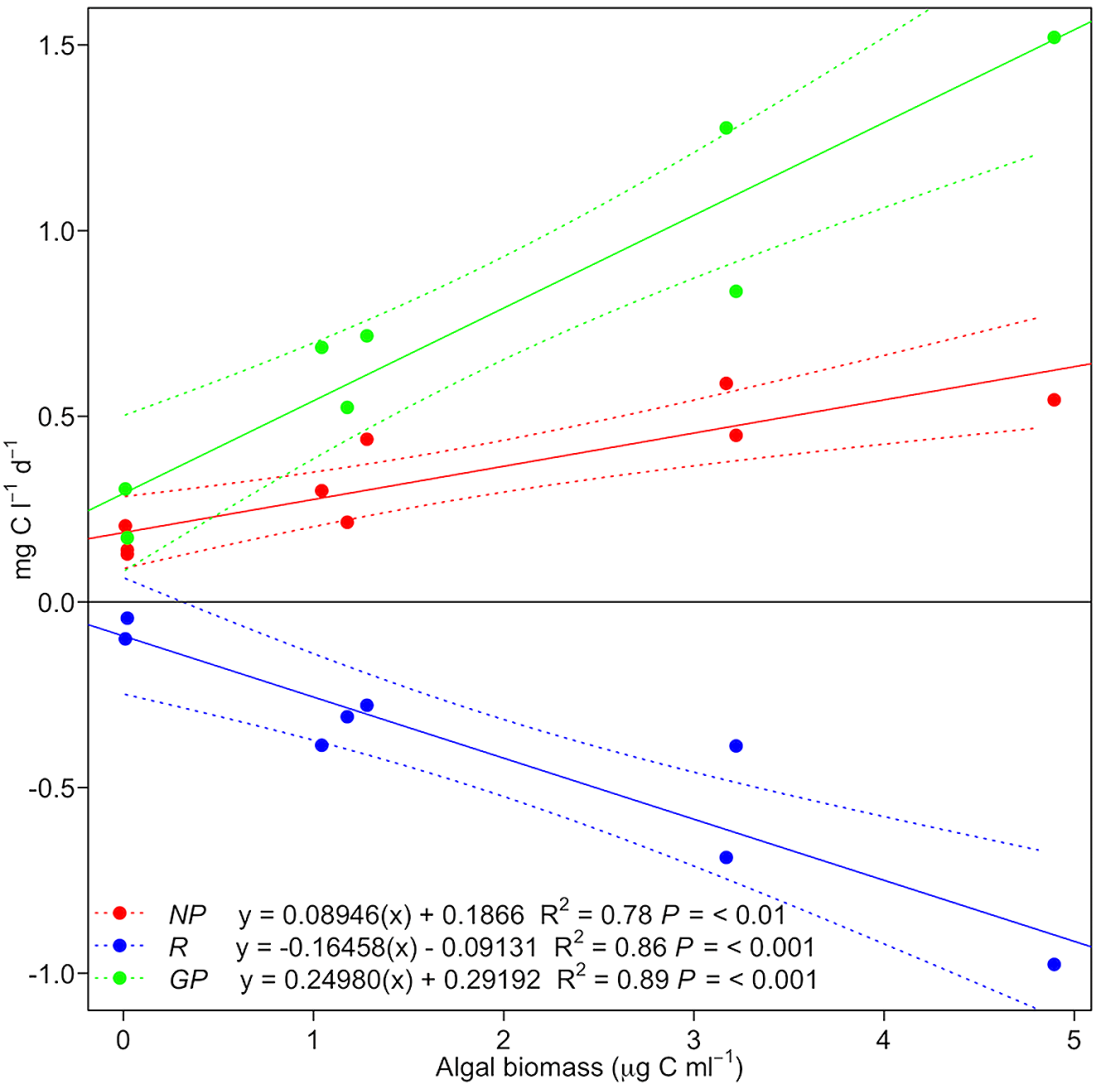
Relationships determined by least-squares linear regression between ice algal biomass and net production (*NP*, red data), respiration (*R*, blue data) and gross production (*GP*, green data), showing regression line (sold lines) and 95% confidence intervals (dashed lines).

Assessment of net production (*NP*) and respiration (*R*) of surface ice containing a high, medium, or low biomass of ice algae demonstrated consistent net autotrophy for surface ice samples (Fig. 9), with significant linear relationships identified between ice algal biomass and *NP*, *GP* and *R* (Fig. 10). Data were highly comparable to rates of primary production assessed using 1 h ^14^C-uptake incubations with ice containing dense algal coverage at a marginal south-western GrIS location (∼1.03 ± 0.62 mg C l^−1^d^−1^, Yallop *et al*. 2012), and values reported from 24 h incubations with ‘dirty-ice’ from Leverett Glacier (∼0.35–1.12 mg C l^−1^ d^−1^, Musilova *et al*. 2017), and the same location as the present study (*NP* = 0.40 ± 0.20, *GP* = 0.64 ± 0.31, *R* = −0.24 ± 0.17 mg C l^−1^ d^−1^, Chandler *et al*. 2015); though no assessment of algal abundance was undertaken during the latter two studies. *GP* was approximately double *R* across all samples (*GP/R* = 2.3 ± 0.2), supporting assumptions built into previous modelling approaches (Cook *et al*. 2012), with an average biomass doubling time of 3.75 ± 0.36 days estimated from *NP* incubations (biomass/*NP*), highly comparable to the 5.5 ± 1.7 days determined by Stibal *et al*. (2017a). Interestingly, doubling times estimated here increased with algal biomass, from 0.11 ± 0.03 days for low biomass ice, to 7.18 ± 1.04 days for high biomass ice, indicating potential resource limitation as algal biomass increases within surface ice. We note, however, the possibility for increased ‘bottle-effects’ with increasing algal biomass within our incubations (see Telling *et al*. 2010), potentially driving our findings toward more conservative estimates as biomass increases. Taken together, data highlight the importance of ice algal communities in driving net autotrophy of GrIS surface habitats, and the potential importance of ice algal blooms in global carbon cycles (see below).

**Figure 11.**
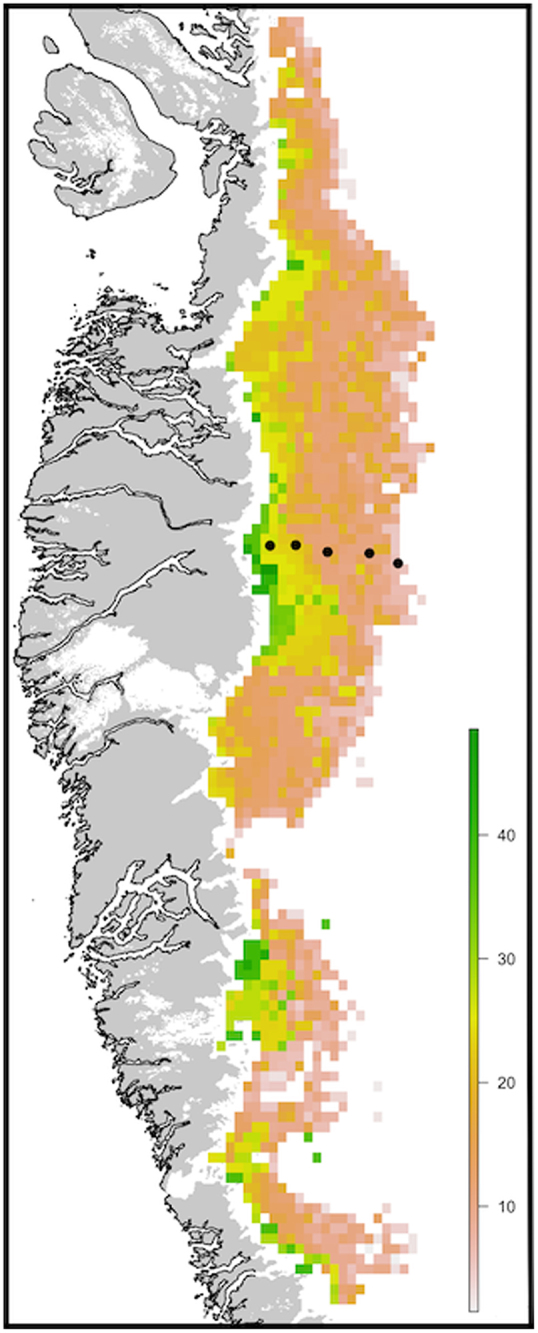
Total ice algal net production potential (kg C km^−2^) estimated for the 2016 ablation season within south-western Greenland. Black dots represent sampling sites of the present study where field measurements were performed (see Fig. 1).

Total ice algal organic carbon accumulation over the 2016 ablation period was estimated as 1.306 Gg C across our ∼8.24 × 10^4^ km^2^ model region, averaging ∼15.82 ± 8.14 kg C km^2^ (Fig. 11). Strong spatial patterning in carbon accumulation was evident, with total *NP* increasing toward the ice margin in concert with biomass increases through time, and exceptional *NP* approaching ∼40–50 kg C km^2^ in this region. In contrast, surface ice located toward the equilibrium line demonstrated total ice algal *NP* potential of <10 kg C km^2^ over the 2016 ablation period, reflecting the shorter duration of bare ice exposure apparent in these locations, and thus reduced capacity to accumulate ice algal biomass. Previously, Cook *et al*. (2012) modelled the *NP* potential of supraglacial algae for a 1600 km^2^ region of the south-western GrIS over the period 2000–2010, reporting an average total *NP* of 16.40 ± 12.80 Gg C for their 1600 km^2^ model region (∼10 250 kg C km^2^, Model 4). As comparison, average *NP* of 0.0022 ± 0.0026 Gg C was reported for carbon accumulation driven by cryoconite alone (∼1.37 kg C km^2^, Model 3, Cook *et al*. 2012). The values determined by the present study were thus significantly reduced as compared to the outputs of Model 4 of Cook *et al*. (2012), though consistent with the expectation that ice algal assemblages provide a greater contribution to carbon accumulation than cryoconite-associated assemblages in supraglacial environments, with our estimates for ice algae ∼11-times those reported for cryoconite alone (Model 3, Cook *et al*. 2012). Given that Model 4 of Cook *et al*. (2012) did not allow for temporal variability in ice algal coverage during ablation seasons, we contend that the organic carbon accumulation determined here for the 2016 ablation period provides a more realistic estimate of the *in-situ* carbon fixation potential of ice algal assemblages across the bare ice zone.

## CONCLUSIONS

Despite their potential importance for surface albedo and carbon flux estimates (Yallop *et al*. 2012; Stibal *et al*. 2017a), few studies have characterised blooms of ice algae that occur in GrIS supraglacial environments. Our results serve to demonstrate the nature of ice algal bloom development, highlighting the capacity for significant increases in algal biomass, associated pigmentation, and carbon fixation potential following snow line retreat. Strengthening of the patterns described here would be anticipated into the future if global temperature increases translate into longer ablation periods, which in turn would expose a greater extent of bare ice in which algal blooms can occur. This process may be further exacerbated by increased deposition of bio-available nutrient resources driven by increased anthropogenic activities. Outcomes of the present study coupled with future research into the mechanisms underlying ice algal pigment regulation and abiotic/biotic controls on biomass within surface ice are required to facilitate projections of the magnitude and impacts of future GrIS ice algal blooms.
